# Eplerenone attenuates pathological pulmonary vascular rather than right ventricular remodeling in pulmonary arterial hypertension

**DOI:** 10.1186/s12890-018-0604-x

**Published:** 2018-03-02

**Authors:** Mario Boehm, Nadine Arnold, Adam Braithwaite, Josephine Pickworth, Changwu Lu, Tatyana Novoyatleva, David G. Kiely, Friedrich Grimminger, Hossein A. Ghofrani, Norbert Weissmann, Werner Seeger, Allan Lawrie, Ralph T. Schermuly, Baktybek Kojonazarov

**Affiliations:** 1 Universities of Giessen and Marburg Lung Center (UGMLC), Excellence Cluster Cardio-Pulmonary System (ECCPS), Member of the German Center for Lung Research (DZL), Aulweg 130, 35392, Giessen, Germany; 2 Department of Infection, Immunity and Cardiovascular Disease, University of Sheffield, Sheffield, UK; 3 Sheffield Pulmonary Vascular Disease Unit, Royal Hallamshire Hospital, Sheffield, UK

**Keywords:** PAH, Eplerenone, Right ventricle

## Abstract

**Background:**

Aldosterone is a mineralocorticoid hormone critically involved in arterial blood pressure regulation. Although pharmacological aldosterone antagonism reduces mortality and morbidity among patients with severe left-sided heart failure, the contribution of aldosterone to the pathobiology of pulmonary arterial hypertension (PAH) and right ventricular (RV) heart failure is not fully understood.

**Methods:**

The effects of Eplerenone (0.1% Inspra® mixed in chow) on pulmonary vascular and RV remodeling were evaluated in mice with pulmonary hypertension (PH) caused by Sugen5416 injection with concomitant chronic hypoxia (SuHx) and in a second animal model with established RV dysfunction independent from lung remodeling through surgical pulmonary artery banding.

**Results:**

Preventive Eplerenone administration attenuated the development of PH and pathological remodeling of pulmonary arterioles. Therapeutic aldosterone antagonism – starting when RV dysfunction was established - normalized mineralocorticoid receptor gene expression in the right ventricle without direct effects on either RV structure (Cardiomyocyte hypertrophy, Fibrosis) or function (assessed by non-invasive echocardiography along with intra-cardiac pressure volume measurements), but significantly lowered systemic blood pressure.

**Conclusions:**

Our data indicate that aldosterone antagonism with Eplerenone attenuates pulmonary vascular rather than RV remodeling in PAH.

## Background

Pulmonary arterial hypertension (PAH) is a devastating disorder characterized by aberrant remodeling of pulmonary arteries that results in sustained pulmonary vasoconstriction, progressively increases pulmonary vascular resistance (PVR) and right ventricular (RV) afterload [1–3]. The persistent increase in afterload maintains high shear stress on the RV myocardium and leads to structural RV remodeling. Current interventions approved for PAH therapy consist of vasodilators that relieve the pulmonary vasoconstrictive component of the disease while the underlying pathological lung and heart remodeling progresses. Therefore, future treatment strategies need to go beyond vasodilation by targeting maladaptive remodeling processes in both, pulmonary vasculature and RV myocardium.

An accumulating body of evidence suggests that dysregulation of the Renin-Angiotensin-Aldosterone-System (RAAS) contributes to the pathogenesis of PAH [4–7]. In particular, the potential contribution of aldosterone – a mineralocorticoid hormone critically involved in systemic blood pressure regulation – to PAH pathogenesis has recently drawn attention. Elevated levels of circulating aldosterone were found in PH patients and correlate with key cardio-pulmonary indices [8]. In line, PH rat models demonstrate increased plasma and lung tissue aldosterone concentrations that correlate with cardio-pulmonary hemodynamics as well as pulmonary vascular remodeling [8, 9] pointing towards a causative role for aldosterone signaling in mediating adverse lung remodeling as seen in PAH development. Pharmacological aldosterone antagonism by the FDA approved drugs Spironolactone or Eplerenone, respectively, directly reduced the pathologic pulmonary vascular remodeling in PAH animal models [10].

On a cellular level, it was demonstrated that aldosterone induces oxidative stress, endothelial dysfunction, inflammation and fibrosis within the pulmonary vasculature. In pulmonary artery smooth muscle cells (PASMCs), aldosterone promotes proliferation, viability and apoptosis resistance [10, 11]. In pulmonary artery endothelial cells (PAECs), aldosterone activates oxidant stress signaling pathways that decrease the bioavailability of the vasodilator nitric oxide, increases inflammation, promotes fibrosis and increases cell proliferation and migration [9, 12, 13]. In PAH, both PASMCs and PAECs are considered key cell types, whose aberrant activation is thought to drive maladaptive remodeling of the pulmonary vasculature.

Aldosterone activation is also associated with decreased diuresis which elevates blood volume and thereby blood pressure. Thus, by closely monitoring electrolytes (sodium/fluid retention and potassium/magnesium wasting), diuretics in combination with aldosterone antagonists effectively reduce blood volume, thereby cardiac load and thus decrease RV wall stress in patients with severe left heart failure [14]. Retrospectively, Spironolactone and Eplerenone therapy have shown direct beneficial effects on the RV in patients with PAH [15]. However, in an experimental rat model of RV failure independent from afterload, RAAS inhibition with Losartan (angiotensin II receptor blocker) and Eplerenone had no direct effects on either RV structure or function [16].

Eplerenone itself is a small molecule suggested to selectively compete with aldosterone for mineralocorticoid receptor binding and as compared with the nonselective mineralocorticoid receptor antagonist Spironolactone has lower affinity for progesterone and androgen receptor binding which is associated with drug-induced gynecomastia, breast pain and impotence [17]. Eplerenone is clinically FDA approved for the treatment of left-sided heart failure and systemic hypertension [18].

Taken together, clinical and experimental data suggest that increased circulating aldosterone levels contribute to the pathogenesis of PAH. To test this hypothesis, we investigated whether pharmacological aldosterone antagonism with Eplerenone attenuates pathological remodeling of the lung and the RV in experimental mouse models. In order to differentiate afterload-dependent from direct myocardial effects, we utilized the SuHx and a pulmonary artery banding (PAB) mouse model.

## Methods

All experiments were performed according to the institutional guidelines that comply with national and international regulations (EU directive 2010/63). The local authorities for animal research approved the study protocol (Regierungspräsidium Giessen, Germany, Gi 32/2013 and The UK Home Office under PPL 40/3517).

### Animal models

Adult male C57BL/6 J mice were purchased from Charles River Laboratories (Sulzfeld, Germany and United Kingdom) and housed under controlled conditions with free access to rodent chow and tap water.

Mice were kept for three weeks under normobaric hypoxia (~ 10% O_2_) and were concomitantly given an injection of sugen5416 (20 mg/kg dissolved in MCC, Tocris) once per week subcutaneously. All animals were randomly assigned for either placebo (standard diet, hereafter referred to as SuHx) or Eplerenone therapy (hereafter referred to as Epl) starting on day one for additional three weeks. Effective dosing was estimated according to prior reports demonstrating efficacy of 200 mg/kg Eplerenone administration [19, 20], wherefore 50 mg Inspra® tablets were homogenized and mixed into standard rodent chow (Altromin, Lage, Germany) to receive a final concentration of 0.1% Eplerenone mixed in chow. Based on prior uptake data, this concentration was estimated to result in ~ 200 mg/kg/d Eplerenone. Control animals were kept under normobaric conditions without sugen5416 injections and were fed standard diet for the entire study period (hereafter referred to as cntrl). Maintained pressure overload was surgically induced by pulmonary artery banding (PAB) as described before [21–23]. PAB-challenged animals were randomly assigned for either placebo (hereafter referred to as PAB) or Eplerenone (0.1% mixed in chow, hereafter referred to as Epl) therapy starting one week after disease commencement for additional two weeks. Control animals underwent the identical surgical procedure without pulmonary artery clipping and were fed control diet for the entire study period (hereafter referred to as sham).

### Heart function assessment

All animals underwent non-invasive transthoracic imaging under continuous isoflurane anesthesia (1.5–2%) to measure RV cardiac output (CO), RV internal diameter (RVID), tricuspid annular plane systolic excursion (TAPSE) and myocardial performance index (MPI) in a blinded manner as described before [24, 25]. Total pulmonary resistance index (TPRi) was calculated as RV systolic pressure divided by echocardiographically determined CI. Subsequently, intra-cardiac catheterization was performed in all animals as previously reported [21, 26]. Terminally, all mice were euthanized by exsanguination and the RVs dissected for tissue weight measurements.

### Histomorphology

Formalin fixed tissue samples were dehydrated, paraffin embedded, sectioned (5 μm) and stained for Alcian Blue Elastic van Gieson (ABVEG), alpha smooth muscle actin (αSMA) and von Willebrand factor (vWF) or picrosirius red and wheat germ agglutinin (WGA) as described before [22, 26, 27].

### Gene expression analysis

Total mRNA was extracted from frozen mouse RV tissues, subsequently transcribed into cDNA and qPCR was performed. Intron-spanning mouse-specific primers for mineralocorticoid receptor (5’-CCGAGATCGTGTATGCAGGC-3′ and 5’-CGCACGAACTGAAGGCTGAT-3′), Collagen 1A1 (Col1A1; 5’-CCGGCTCCTGCTCCTCTTAG-3′ and 5’-CCTCGGGTTTCCACGTCTCA-3′), Collagen 3A1 (Col3A1; 5’-CCAGGAGCCAGTGGCCATAA-3′ and 5’-GGGGCACCAGGAGAACCATT-3′) and porphobilinogen deaminase (PBGD; 5′-GGGAA CCAGCTCTCTGAGGA-3′ and 5’-GAATTCCTGCAGCTCATCCA-3′) were designed using sequence information from the NCBI database and were purchased from Metabion (Martinsried, Germany). Target gene Ct values were normalized to the housekeeping gene PBGD and expression was calculated as percentage of sham controls.

### Data analysis

All data are presented as mean ± SD analyzed with one-way ANOVA followed by Newman-Keuls multiple comparison post-hoc test. Differences were considered statistically significant for *p* < 0.05.

## Results

### Eplerenone attenuates SuHx-induced PH and pulmonary vascular remodeling

**Fig. 1:**
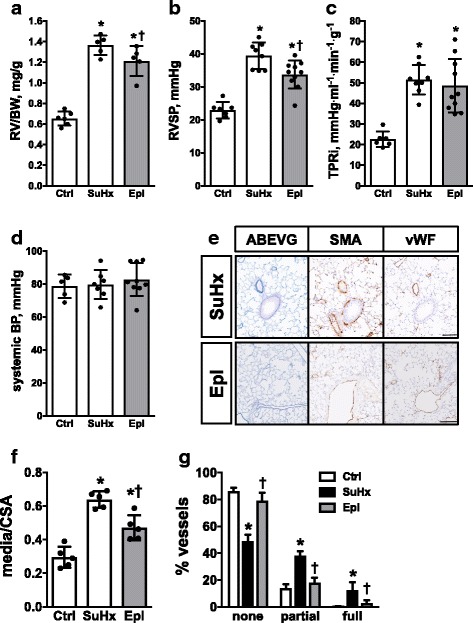
Eplerenone attenuates SuHx-induced PH and pulmonary vascular remodeling. Preventive Eplerenone administration (Inspra®, 0.1% mixed in chow) to mice attenuated the development of SuHx-induced RV hypertrophy (RV/BW, mg/g; **a**) and increased RV systolic pressure (RVSP, mmHg; **b**). Total pulmonary resistance index (TPRi, ml^− 1^∙min^− 1^∙g^− 1^; **c**) and systemic blood pressure (systemic BP, mmHg; **d**) remained unaltered by Eplerenone administration. Representative lung sections stained for Alcian Blue Elastic van Gieson (ABEVG), smooth muscle actin (SMA) and von Willebrand factor (vWF) are shown in **e**. Medial hypertrophy is presented as ratio of media to cross sectional area [CSA] (**f**). Remodeling of pulmonary blood vessels is demonstrated by muscularization (percentage of vessels). *n* = 5–10 mice per group; *: *p* < 0.05 vs. cntrl; †: *p* < 0.05 vs. SuHx

Oral Eplerenone administration (0.1% mixed in chow) for three consecutive weeks significantly attenuated the development of PH in the SuHx mouse model (Sugen-5416 injection followed by chronic hypoxia) demonstrated by reduced RV hypertrophy (1.2 ± 0.1 vs. 1.4 ± 0.1, *p* < 0.05) (RV/BW, Fig. 1a) and reduced RV systolic pressure (33.8 ± 4.3 vs. 39.5 ± 4.0 mmHg, *p* < 0.05) (RVSP, Fig. 1b) as compared with controls. Total pulmonary resistance index tended to decrease upon Eplerenone administration (48.6 ± 13.0 vs. 51.4 ± 7.2 mmHg∙ml^− 1^∙min^− 1^∙g^− 1^, *p* > 0.05) (TPRi, Fig. 1c) without affecting systemic blood pressure (82.8 ± 10.0 vs. 79.7 ± 8.8 mmHg, *p* > 0.05) (systemic BP, Fig. 1d) indicating a selective direct effect on the diseased pulmonary circulation. Reduced remodeling of pulmonary arteries was demonstrated by histomorphology (Fig. 1e) and quantified as medial hypertrophy (0.47 ± 0.08 vs. 0.64 ± 0.05, *p* < 0.05) (media/cross sectional vessel area, Fig. 1f) and percentage of muscularized pulmonary arterioles (Fig. 1g).

### Eplerenone therapy reduces systemic blood pressure upon chronic RV pressure overload without direct effects on RV structure or function

**Fig. 2:**
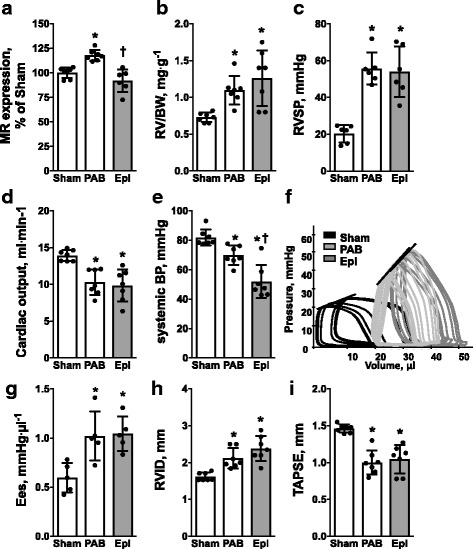
Eplerenone reduces systemic blood pressure without direct effects on RV structure and function upon PAB. Eplerenone therapy normalizes RV mineralocorticoid receptor gene expression (MR expression, percentage of sham; **a** without affecting RV hypertrophy (RV/BW, mg/g; **b** RV systolic pressure (RVSP, mmHg; **c** or cardiac output (ml∙min^− 1^, **d** while systemic blood pressure (systemic BP, mmHg; **e** dropped down significantly. Representative pressure-volume loops with lined end-systolic elastances (**f**). Quantification of end-systolic elastance (Ees, mmHg∙μl; **g**), echocardiography-derived RV internal diameter (RVID, mm; **h**) and Tricuspid annular plane systolic excursion (TAPSE, mm; **i**) reveal no difference between PAB and Eplerenone treated mice. *n* = 5–7 mice per group; *: *p* < 0.05 vs. cntrl; †: *p* < 0.05 vs. PAB

Therapeutic efficacy of Eplerenone – starting after one week when animals in both PAB groups showed comparable signs of established RV dysfunction (data not shown) – normalized mineralocorticoid receptor gene expression in the RV (92 ± 12% vs. 118 ± 8%, *p* < 0.05) (Fig. 2a) without affecting RV hypertrophy (1.3 ± 0.4 vs. 1.1 ± 0.2, *p* > 0.05) (RV/BW, Fig. 2b), RV systolic blood pressure (54.0 ± 13.7 vs. 55.7 ± 8.7 mmHg, *p* > 0.05) (RVSP, Fig. 2c) or cardiac output (9.8 ± 2.2 vs. 10.3 ± 1.8 ml∙min^− 1^, *p* > 0.05) (Fig. 2d) as compared with placebo controls, demonstrating no direct cardioprotective effects of Eplerenone on the RV. Importantly, systemic blood pressure dropped down (52.0 ± 11.3 vs. 69.8 ± 6.5 mmHg, *p* < 0.05) (Fig. 2e) indicating effective dosing and a significant systemic impact of Eplerenone on the stressed heart. Pressure-volume analysis (Fig. 2f) demonstrated unchanged contractility by RV end-systolic elastance (1.04 ± 0.08 vs. 1.02 ± 0.11 mmHg∙μl^− 1^, *p* > 0.05) (Ees, Fig. 2g). In addition, echocardiography revealed no change in RV dilatation (unchanged RV internal diameter (2.4 ± 0.1 vs. 2.1 ± 0.3 mm, *p* > 0.05) (RVID, Fig. 2h)) and tricuspid annular plane systolic excursion (1.0 ± 0.1 vs. 1.0 ± 0.1 mm, *p* > 0.05) (TAPSE, Fig. 2i) upon Eplerenone therapy as compared with placebo controls.

**Fig. 3:**
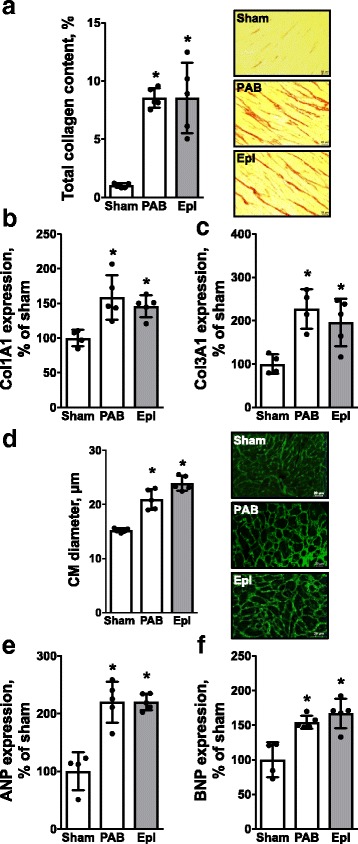
Eplerenone has no direct effect on pressure overload-induced structural RV remodeling. Pharmacological aldosterone antagonism with Eplerenone had no effect on RV total collagen content assessed by picrosirius red stains (percentage of the total RV; **a**), *Col1A1* (percentage of sham; **b**) and *Col3A1* gene expression (percentage of sham; **c**). Eplerenone did not affect cardiomyocyte hypertrophy (CM diameter, μm; **d**), ANP (**e**) or BNP gene expression (percentage of sham; **f**). *n* = 4–5 mice per group; *: *p* < 0.05 vs. cntrl

Furthermore, chronic pressure overload-induced structural RV adaptation remained unaltered by Eplerenone therapy as total RV collagen content (8.6 ± 1.4 vs. 8.5 ± 0.4%, *p* > 0.05) (Fig. 3a), *Col1A1* (147 ± 7 vs. 159 ± 14%, *p* > 0.05) (Fig. 3b) and *Col3A1* (196 ± 25 vs. 227 ± 23%, *p* > 0.05) (Fig. 3c) gene expression were not affected. Also, pharmacological aldosterone antagonism had no effect on cardiomyocyte hypertrophy (23.9 ± 0.6 vs. 20.9 ± 0.8 μm, *p* > 0.05) (CM diameter, Fig. 3d) and heart failure marker gene expression (221 ± 6 vs. 220 ± 16%, *p* > 0.05 for ANP, Fig. 3e and 167 ± 10 vs. 154 ± 4%, *p* > 0.05 for BNP, Fig. 3f).

## Discussion

The current study demonstrates that oral administration of an aldosterone receptor antagonist attenuates maladaptive remodeling of the pulmonary vasculature without direct effects on RV structure and function in rodent models. Eplerenone decreased SuHx-induced remodeling of small pulmonary arteries without affecting the systemic circulation. In a PAB mouse model, Eplerenone induced systemic hypotension in animals with established RV dysfunction - most likely due to cardiac unloading - while RV structure and function remained unaffected, demonstrating no direct cardioprotective effects independent from afterload. Taken together, these data suggest that aldosterone plays a pathological role in maladaptive remodeling of the pulmonary vasculature rather than the RV. The therapeutic effect of Eplerenone on the diseased pulmonary circulation, however, remains to be characterized (including the effects of Eplerenone on PASMCs and PAECs).

The development of PH in mice following vascular endothelial growth factor receptor (VEGFR) blockade and chronic hypoxia (SuHx model) was effectively attenuated by concomitant treatment with the oral available aldosterone antagonist Eplerenone (Inspra®, 0.1% mixed in standard chow) through reduced remodeling of small pulmonary arteries. Eplerenone was oral available and efficacious by decreasing RV systolic pressure and hypertrophy without affecting the systemic circulation - showing a direct effect on the pulmonary circulation. We estimated 0.1% Eplerenone mixed in chow to result in ~ 200 mg/kg/d effective dosing, confirmed by PH attenuation in the SuHx model, while an exact free plasma concentration for Eplerenone is not available, may vary between animals and is a clear limitation to this study. Though, a pathological role for aldosterone in maladaptive remodeling of pulmonary arteries has previously been described in PH rat models. In monocrotaline-induced PH, pharmacological aldosterone antagonism with either Spironolactone or Eplerenone decreased vascular hyperplasia and vessel thickening [10, 11]. Also, aldosterone antagonism has been shown to reduce neointimal hyperplasia in rats with SuHx-induced PH [10]. These data are consistent with the decreased pulmonary vascular remodeling observed in the current study, which show that preventive Eplerenone administration reduced vascular thickening. On a cellular level, previous studies have demonstrated that aldosterone activates an Akt/mTOR/Raptor axis that promotes pulmonary artery smooth muscle cell (PASMC) proliferation, cell migration, viability and apoptosis resistance [10, 11]. It was shown that chronic hypoxia itself selectively induces aldosterone synthesis autonomously in pulmonary artery endothelial cells (PAECs) by upregulation of the steroidogenic acute regulator protein (StAR). Elevated aldosterone levels were linked to vasoconstriction by PAEC-derived endothelin 1 release, reactive oxidant signaling, reduced nitric oxide bioavailability and fibrosis [9, 12, 13]. In PAH, both PASMCs and PAECs are considered key cell types whose aberrant activation leads to pulmonary vascular remodeling that results in a sustainably increased RV afterload.

Therapeutic strategies for the treatment of PAH aim to halt or even reverse maladaptive lung remodeling thereby reducing RV afterload, wall stress, myocardial oxygen consumption, and ischemia to improve the contractile state of the heart. Currently, RV afterload and wall stress reduction in PAH is achieved through pulmonary vasodilation [28]. Extensive work from the field of left-sided heart failure has already revealed that in addition to vasodilation, cardiac unloading through blood volume reduction via RAAS inhibition beneficially affects cardiac structure and function [14, 17]. In line, several reports link dysfunctional RAAS activation to PAH pathogenesis [4, 5, 7]. However, experimental and clinical data demonstrate that RAAS blockade has no or only minor direct beneficial effects on the RV myocardium despite systemic unloading, suggesting that the response to RAAS therapy might be critically different between the LV and the RV [16, 29, 30]. The current study extends these observations into oral administration of Eplerenone in mice with isolated RV pressure overload independent from afterload in a dosage that has proven efficacy in attenuating pathologic pulmonary vascular remodeling. Beneficial effects observed in PH animal models might be secondary due to afterload reductions. Here, Eplerenone therapy starting when RV dysfunction was established had no direct effect on the RV myocardium while systemic blood pressure dropped down. Interestingly, Eplerenone lowered the systemic blood pressure only when administered to animals with established RV dysfunction but not in the preventive SuHx model, pointing towards a differential role in the stressed RV which might be more vulnerable to aldosterone antagonism. Similar observations were made in a study addressing the effects of RAAS inhibition on the RV in a chronic pressure overload rat model [16].

A clinical role for Eplerenone has been described in cardiovascular protection, was confirmed in several clinical trials in patients with left-sided heart failure (RALES, EPHESUS, ENPHASIS-HF) and has been extensively characterized in models of LV remodeling [14, 31, 32]. However, the molecular mechanisms are not fully understood. A growing body of evidence suggests that Eplerenone mediates its effects in part through competing with aldosterone for mineralocorticoid receptor binding – however, the mechanistic insights how Eplerenone might affect mineralocorticoid receptor expression, as it was observed in this study, are still elusive. In heart failure mouse models, genetic inactivation of the mineralocorticoid receptor signaling pathway improved LV function [33]. Specifically, cardiomyocyte but not fibroblast restricted mineralocorticoid receptor deficiency improved LV function and reduced LV dilation upon trans-aortic constriction pointing towards a key role for cardiomyocyte mineralocorticoid receptor signaling in the pathogenesis of left heart failure [34]. In line, Eplerenone therapy in wildtype animals with heart failure improved LV function and reduced LV dilation [35]. Whether elevated cardiac mineralocorticoid receptor signaling is a disease consequence or drives heart failure progression is not clear. In the current study, we report increased mineralocorticoid receptor gene expression directly in the hypertrophied RV – suggesting increased mineralocorticoid receptor signaling - which was normalized by Eplerenone therapy without functional or structural RV improvements. These data show that increased mineralocorticoid receptor activation is rather a disease consequence than a driver of RV heart failure pathogenesis.

## Conclusions

In summary, this study reports a benefit of pharmacological aldosterone antagonism with Eplerenone in PAH by directly targeting the pulmonary vasculature while further studies are warranted to fully characterize the therapeutic benefit of Eplerenone on the diseased pulmonary vasculature and dissect the mechanistic role of aldosterone in PAH pathophysiology. The clinical relevance of aldosterone antagonism as a therapeutic approach for PAH is currently being evaluated in clinical trials. Patients are recruited for a Phase 2, dose-ranging, randomized, placebo controlled study (→→→→ClinicalTrials.gov identifier: NCT01712620) that is designed to compare the effectiveness of Spironolactone in treating PAH versus placebo. By targeting the maladaptive pulmonary vascular remodeling processes, that current PAH therapies do not, aldosterone antagonism with Spironolactone is expected to improve exercise capacity and endothelial dysfunction in PAH. To find out whether an enhanced cardiopulmonary fitness (exercise capacity and RV function) improves the quality of life, Spironolactone in combination with an endothelin receptor type A blocker will be administered to PAH patients with a LV ejection fraction > 50% in a prospective, double blind, placebo-controlled phase 4 study (ClinicalTrials.gov identifier: NCT02253394).
