# Correction to: Eplerenone attenuates pathological pulmonary vascular rather than right ventricular remodeling in pulmonary arterial hypertension

**DOI:** 10.1186/s12890-022-01978-0

**Published:** 2022-07-20

**Authors:** Mario Boehm, Nadine Arnold, Adam Braithwaite, Josephine Pickworth, Changwu Lu, Tatyana Novoyatleva, David G. Kiely, Friedrich Grimminger, Hossein A. Ghofrani, Norbert Weissmann, Werner Seeger, Allan Lawrie, Ralph T. Schermuly, Baktybek Kojonazarov

**Affiliations:** 1 Universities of Giessen and Marburg Lung Center (UGMLC), Excellence Cluster Cardio-Pulmonary System (ECCPS), Member of the German Center for Lung Research (DZL), Aulweg 130, 35392 Giessen, Germany; 2 Department of Infection, Immunity and Cardiovascular Disease, University of Sheffield, Sheffield, UK; 3 Sheffield Pulmonary Vascular Disease Unit, Royal Hallamshire Hospital, Sheffield, UK

## Correction to: BMC Pulmonary Medicine (2018) 18:41 https://doi.org/10.1186/s12890-018-0604-x

Following publication of the original article [1], it was brought to the authors’ attention that representative histological images in Fig. 3D had been erroneously duplicated from two other articles [2, 3].

**Fig. 3:**
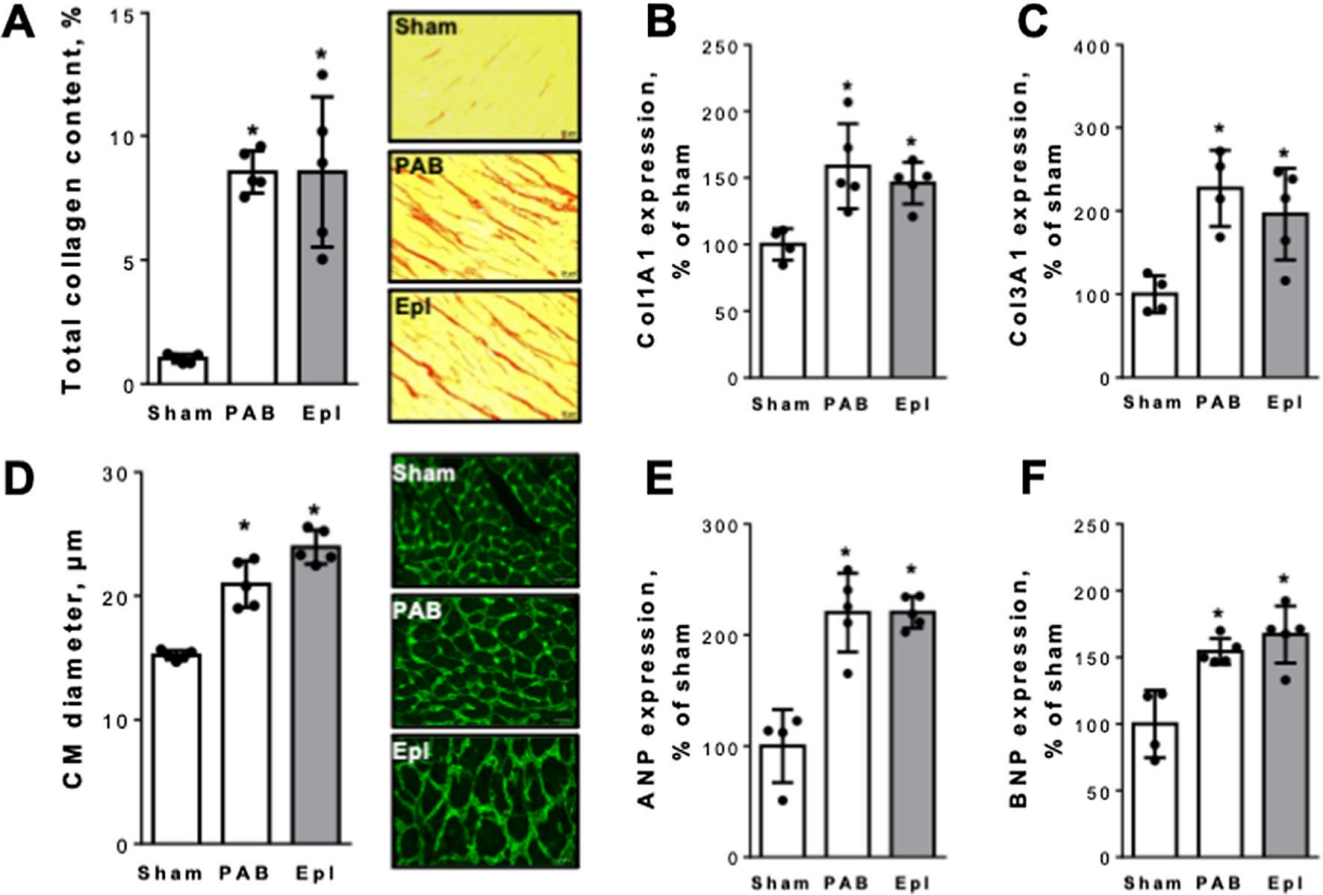
Eplerenone has no direct effect on pressure overload-induced structural RV remodeling. Pharmacological aldosterone antagonism with Eplerenone had no effect on RV total collagen content assessed by picrosirius red stains (percentage of the total RV; **A**), Col1A1 (percentage of sham; **B**) and Col3A1 gene expression (percentage of sham; **C**) Eplerenone did not affect cardiomyocyte hypertrophy (CM diameter, μm; **D**), ANP (**E**) or BNP gene expression (percentage of sham; **F**). n = 4–5 mice per group; *p < 0.05 vs. cntrl

The figure has been corrected in the published article and the correct figure is shown in this correction. This correction does not affect the results or conclusion of the article.

The authors apologize for any inconvenience caused.
